# Linking replication stress with heterochromatin formation

**DOI:** 10.1007/s00412-015-0545-6

**Published:** 2015-10-28

**Authors:** Ivaylo Nikolov, Angela Taddei

**Affiliations:** Institut Curie, PSL Research University, Paris, F-75248 France; CNRS, UMR 3664, Paris, France; Sorbonne Universtiés, UPMC Univ Paris, Paris, France

**Keywords:** Heterochromatin, Gene silencing, Replication stress, Epigenetics

## Abstract

The eukaryotic genome can be roughly divided into euchromatin and heterochromatin domains that are structurally and functionally distinct. Heterochromatin is characterized by its high compaction that impedes DNA transactions such as gene transcription, replication, or recombination. Beyond its role in regulating DNA accessibility, heterochromatin plays essential roles in nuclear architecture, chromosome segregation, and genome stability. The formation of heterochromatin involves special histone modifications and the recruitment and spreading of silencing complexes that impact the higher-order structures of chromatin; however, its molecular nature varies between different chromosomal regions and between species. Although heterochromatin has been extensively characterized, its formation and maintenance throughout the cell cycle are not yet fully understood. The biggest challenge for the faithful transmission of chromatin domains is the destabilization of chromatin structures followed by their reassembly on a novel DNA template during genomic replication. This destabilizing event also provides a window of opportunity for the de novo establishment of heterochromatin. In recent years, it has become clear that different types of obstacles such as tight protein-DNA complexes, highly transcribed genes, and secondary DNA structures could impede the normal progression of the replisome and thus have the potential to endanger the integrity of the genome. Multiple studies carried out in different model organisms have demonstrated the capacity of such replisome impediments to favor the formation of heterochromatin. Our review summarizes these reports and discusses the potential role of replication stress in the formation and maintenance of heterochromatin and the role that silencing proteins could play at sites where the integrity of the genome is compromised.

In eukaryotic cells, the genetic information is stored as a complex of DNA and proteins called chromatin. Based on cytological observations, the German botanist Emil Heitz distinguished the following two types of chromatin: euchromatin, corresponding to regions of mitotic chromosome that decondensed during interphase, and heterochromatin, corresponding to regions that remained condensed throughout the whole cell cycle. Heitz proposed that heterochromatin reflects a functionally inactive state of the genome. Heterochromatin was subsequently found to be associated with gene repression and specific patterns of post-translational histone modifications or chromatin marks that recruit general repressors of transcription. Apart from their role in transcriptional repression, heterochromatic regions are also an important determinant of the spatial organization of the genome. They tend to cluster together and localize close to the nuclear periphery or around nucleoli, forming functional nuclear subcompartments, which sequester the pool of silencing proteins (Padeken and Heun 2014; Meister and Taddei 2013; Saksouk et al. 2015).

Heterochromatin can be generally divided into two major types—constitutive and facultative—that display different molecular signatures. Constitutive heterochromatin forms at telomeres, centromeres, and repetitive elements, where it plays a major role in genome stability (Dillon 2004; Saksouk et al. 2015). Facultative heterochromatin is developmentally regulated and acts as a key regulator of cellular differentiation and morphogenesis (Trojer and Reinberg 2007).

How heterochromatin domains are formed at specific loci and maintained throughout cellular division is still an open question, but DNA replication appears to be an important player in this process.

The replication process is per se a disrupting event that challenges established patterns of chromatin marks but at the same time provides a window of opportunity to change chromatin status. Cells have thus evolved specific mechanisms to coordinate DNA replication with the re-assembly of a similar chromatin environment on the two daughter strands (Alabert and Groth 2012).

Direct molecular links between replication and heterochromatin factors have been reported in many different species. One striking example is the conserved role of the origin recognition complex (ORC) in heterochromatin formation that was first observed in budding yeast, where the ORC subunit Orc1 interacts directly with the silencing factor Sir1 (Bell et al. 1993). Furthermore, Sir3, which is the structural component of silent chromatin in this species, is a paralog of Orc1 that arose from the whole genome duplication event in the Saccharomycetaceae order (Hickman et al. 2011). It is likely that Sir3 acquired its silencing function after gene duplication, as the Orc1 protein fulfills the role of Sir3 in *Kluyveromyces lactis*, a species that diverged from Saccharomyces prior to Orc1 duplication (Hickman and Rusche 2010). Interestingly, although heterochromatin components are not conserved between budding yeast and metazoans, ORC has been shown to interact with heterochromatin proteins such as heterochromatin protein 1 (HP1) in metazoans, indicating the conserved cross-talk between DNA replication and chromatin state.

Although ORC is present at all replication origins, heterochromatin is formed and confined to specific domains, suggesting that the interaction between ORC and heterochromatic factors is restricted to specific regions or that it leads to heterochromatin formation only under specific circumstances.

What is the hallmark of heterochromatin domains that distinguishes them from euchromatin regions and triggers the formation of heterochromatic structures? One possibility that we will discuss in this review is that these regions are difficult to replicate and that this feature could favor heterochromatin formation. Indeed, it is noteworthy that sequences embedded into heterochromatin are often inherently difficult to replicate and are thus a potential source of replication stress (Ivessa et al. 2003; Miller et al. 2006; Sfeir et al. 2009; Zaratiegui et al. 2011b). Replication stress is defined as slowing or stalling in replication fork progression and arises from many different sources including repetitive sequences, tight DNA-protein complexes, DNA secondary structures, DNA–RNA hybrids (R-loops), collisions between replication and transcription machineries, DNA lesions, or misincorporation of ribonucleotides (Aguilera and Garcia-Muse 2012; Bochman et al. 2012; Tourriere and Pasero 2007). Although replication stress can occur in both euchromatin and heterochromatin, several lines of evidence indicate that replication stress could be an important, although not unique, determinant in the formation and maintenance of heterochromatin states.

In this review, we will try to summarize the current knowledge on the mechanisms that link replication stress with heterochromatin formation and maintenance and discuss the potential function of this link.

## Replicating heterochromatin

As mentioned above, heterochromatin comes in different flavors; here, we will briefly discuss mechanisms involved in pericentric heterochromatin replication that can serve as a paradigm for heterochromatin duplication. In mammalian cells, heterochromatin is formed in pericentric regions via the recruitment of HP1, which exists in the following three isoforms: α, β, and γ (Maison and Almouzni 2004), which have distinct functions in heterochromatin formation depending on the stage of differentiation (Aucott et al. 2008; Caillier et al. 2010; Sridharan et al. 2013). HP1 association with chromatin relies on the activity of the Suv39h1 methyltransferase, which creates a docking site for HP1 by trimethylating H3 lysine 9 (K9) (Maison and Almouzni 2004). HP1 also physically interacts with Suv39h1, providing a feed-forward loop to spread heterochromatin to neighboring nucleosomes.

During DNA replication, nucleosomes disassemble ahead of the fork and parental H3–H4 histones are handled by the histone chaperone anti-silencing function 1 (ASF1), which associates with the replicative DNA helicase (Alabert and Groth 2012). ASF1 is thought to coordinate the recycling of parental histones and de novo deposition of newly synthesized histones on the daughter strands. Chromatin assembly is coupled to DNA synthesis by the interaction between the sliding clamp proliferating cell nuclear antigen (PCNA) and the chromatin assembly factor 1 (CAF1) (Shibahara and Stillman 1999). Additionally, CAF1 interacts with HP1α and the histone lysine N-methyltransferase SetDB1, which monomethylates H3.1 (Loyola et al. 2009). Thus, in addition to its role in depositing H3 onto newly replicated DNA, CAF1 helps to target SetDB1 and HP1α to sites of heterochromatin formation, promoting both the monomethylation of H3.1K9 and the loading of HP1α in these regions. H3K9me1 is subsequently recognized by the methyltransferase Suv39h1, which trimethylates H3K9, thus allowing HP1 binding. Furthermore, recycled parental H3K9me3 also have the potential to recruit HP1 (Rivera et al. 2014). Duplication of HP1-associated chromatin thus appears to be a robust process involving parallel pathways. Similar mechanisms seem to be at play in the transmission of other heterochromatin-associated histone marks such as H4K20me (Rivera et al. 2014). In addition, HP1α is targeted as a sumoylated form to pericentric heterochromatin via its association with non-coding transcripts corresponding to major satellite repeats, independently of the methyltransferase activity of Suv39h1 (Maison et al. 2011). Whether this pathway requires replication is still an open question.

## Heterochromatin protein at DNA damage sites

In response to DNA damage, chromatin undergoes global decondensation. This process has been proposed to facilitate genome surveillance by enhancing the access of DNA damage response (DDR) proteins to sites of damage (Adam et al. 2015). Paradoxically, heterochromatin factors such as KAP1, HP1, and the H3K9 methyltransferase Suv39h1 were shown to accumulate rapidly and transiently at DNA damage sites (Ayrapetov et al. 2014; Lemaitre and Soutoglou 2014). This occurs in both euchromatic and heterochromatic regions, suggesting a general role for this response in DNA repair. Indeed, depletion of two isoforms of HP1 (α and β) impairs homologous recombination (HR) efficiency, whereas depletion of HPγ gamma had the opposite effect, through mechanisms that are still under debate (Lemaitre and Soutoglou 2014; Soria and Almouzni 2013). A recent study showed that the rapid loading of HP1/KAP1/Suv39h1 leads to the trimethylation of H3K9 over tens of kilobase flanking double-strand breaks (DSBs). Although this H3K9me3 is often associated with heterochromatin, the modification of this large region is thought to help recruit and activate the Tip60 acetyltransferase, which recognizes H3K9me3. Tip60 promotes nucleosome turnover and activation of the ataxia telangiectasia mutated (ATM) checkpoint kinase, which acts as a negative feedback by removing the HP1/KAP1/suv39 complex from chromatin (Ayrapetov et al. 2014).

Beside its potential direct impact on HR, heterochromatin-associated proteins might also help coordinate transcription and repair. Indeed, polycomb proteins that are involved in gene silencing at facultative heterochromatin are also recruited to DSB sites, where they are thought to switch off transcription to facilitate DSB repair (Kakarougkas et al. 2014; Ui et al. 2015; Vissers et al. 2012).

HP1 recruitment at sites of DNA damage is again dependent on its interaction with CAF1 (Baldeyron et al. 2011; Loyola et al. 2009), which is recruited to DNA synthesis repair sites by PCNA (Moggs et al. 2000). CAF1 is conserved in budding yeast, where it also contributes to the maintenance of silent chromatin (Enomoto and Berman 1998) and genome stability (Kaufman et al. 1997). Other links between DNA repair and heterochromatin have been reported in budding yeast. Similar to HP1, its functional homologue in budding yeast (Sir3) has been found to localize at sites of DNA damage (Martin et al. 1999; Mills et al. 1999), although the function of this recruitment remains elusive. Furthermore, the Ku protein plays essential roles in protecting DSB against resection and in promoting subtelomeric gene repression (Boulton and Jackson 1996, 1998). More recently, artificially tethering the ATM checkpoint kinase Tel1 or the DNA repair protein Mre11 to a defective silencer was shown to promote silencing at this locus. Tel1 has been shown to interact with Sir2 in the two-hybrid system, as has Mre11 with both Sir3 and Sir4 (Kirkland et al. 2015). Hence, Tel1 and Mre11 may trigger silencing through the direct recruitment of the SIR complex.

Thus, both DDR and replication are linked to the recruitment of silencing proteins in different species, sometimes ultimately leading to chromatin opening. Silencing proteins may also contribute to genome stability, although this has not always been clearly demonstrated.

## Replication stress and senescence-associated heterochromatin foci in mammalian cells

Cellular senescence is a well-studied example of a process during which chromatin undergoes a massive reorganization. Cellular senescence is an irreversible proliferation arrest, thought to contribute to tumor suppression, wound healing, development, and aging (Rai and Adams 2013). Entry into senescence can be triggered by different sources of stress, including telomere shortening (replicative senescence) and oncogene activation (oncogene-induced senescence (OIS)) (Rai and Adams 2013; Serrano et al. 1997). OIS is accompanied by the accumulation and compaction of chromatin into large subnuclear heterochromatic domains, termed senescence-associated heterochromatin foci (SAHFs) (Narita et al. 2003; Zhang et al. 2005). These structures harbor heterochromatic markers, such as H3K9me3, the histone variant macroH2A, high-mobility group A (HMGA), and the three HP1 isoforms (Corpet and Stucki 2014; Narita et al. 2003, 2006; Zhang et al. 2005).

Importantly, SAHF formation is not a universal feature of senescence as it is less prevalent in replicative senescence and is highly variable between cell types. Thus, SAHF formation appears to be influenced by the origin of the stress that triggers senescence (Kosar et al. 2011). Senescence induced by oncogenes arises from an acute stress at stalled replication forks, leading to irreparable DNA damage (Bartkova et al. 2006; Di Micco et al. 2006, 2011). However, DNA breaks per se do not seem to be the source of SAHF formation, as the activation of the S phase checkpoint kinase ataxia telangiectasia and Rad3-related (ATR) protein in the absence of DNA damage is sufficient to cause cellular senescence and appearance of SAHF (Toledo et al. 2008). Interestingly, oncogene-induced SAHF formation depends on DNA replication and ATR (Di Micco et al. 2011). Furthermore, topological and chromatin regulators involved in the DNA replication process, such as topoisomerase 1 and ASF1, are also essential for SAHF formation. Together, these data support a causal link between oncogene-altered DNA replication and SAHF formation (Humbert et al. 2009; Zhang et al. 2005, 2007).

It is still unclear to what extent SAHF plays a role in the tumor suppressive function of senescence in vivo. Heterochromatinization of proliferation genes through SAHF has been proposed to directly contribute to proliferation arrest. Indeed, disruption of the Suv39h1 H3K9 methyltransferase dramatically accelerates Ras-induced T cell lymphomagenesis in a mouse model (Braig et al. 2005). However, SAHF formation is not sufficient to drive proliferation arrest as SAHF persists in p53 and ATM mutants, in which the cell cycle arrest has been relieved (Di Micco et al. 2011; Rai and Adams 2013). In this case, it was proposed that SAHF protects cells expressing oncogenes from undergoing apoptosis by dampening the DNA damage response (Di Micco et al. 2011). In support of this hypothesis, heterochromatin has been reported to modulate DDR activation in different model systems (see above and Murga et al. 2007). Indeed, loss of HP1γ or the Suv39 methyltransferase led to an amplification of the DDR, eventually resulting in cell apoptosis (Di Micco et al. 2011). As several cancer cell lines display elevated levels of H3K9me3 and HP1, it has been proposed that the inactivation of tumor suppressors, such as p53, hijacks SAHFs to maintain DDR at sublethal levels, thus allowing proliferation. In summary, on the one hand, SAHF might contribute to tumor suppression via repression of proliferation-promoting genes such as cyclin A, while on the other hand, SAHF may dampen the DNA damage response, suppress apoptosis, and promote viability. As a consequence, altering heterochromatin might have different outcomes depending on the stage of oncogenic progression and on the history that led to transformation.

## Replication roadblocks and heterochromatin formation

### Tight DNA-protein complexes

Budding yeast does not show condensed chromatin during interphase and lacks most of the molecular markers that typify heterochromatin in most other eukaryotes, such as H3K9me3 and HP1. Instead, repressed chromatin is generated specifically at telomeres and cryptic mating-type loci (*HM*s) by the recruitment of a complex of silent information regulators, namely, Sir2, Sir3, and Sir4 (Kueng et al. 2013; Taddei and Gasser 2012). The complex has the ability to spread along the chromatin fiber from nucleation sites due to the enzymatic activity of Sir2 that deacetylates histone H4 tails from neighboring nucleosomes and to the affinity of Sir3 for deacetylated histones (Hecht et al. 1995; Imai et al. 2000; Rusche et al. 2003; Smith et al. 2000; Tanner et al. 2000). At telomeres, heterochromatin formation is nucleated by Rap1, which binds telomeric TG_1–3_ repeats and interacts with both Sir3 and Sir4 (Konig and Rhodes 1997; Moretti et al. 1994; Oppikofer et al. 2013). At *HM*s, nucleation is dependent on the presence of “silencer” elements. These elements contain combinations of binding sites for a set of factors Rap1, Abf1, and Sum1 and a member of the origin recognition complex (Orc1) (Kueng et al. 2013; Taddei and Gasser 2012). Intriguingly, Rap1 and Abf1 are two of the most common transcription factors in the yeast genome, and Orc1, as already discussed, is a member of the essential ORC complex, important for the firing of origins of replication (Bell and Stillman 1992; Shore 1994; Yarragudi et al. 2007). How the juxtaposition of these factors leads to the nucleation of silencing is still unknown. The current model proposes that the juxtaposition of factors with a low affinity for the SIR complex could generate a high-affinity binding site. However, although Rap1 interacts directly with Sir3 and Sir4 and Orc1 with Sir1, which in turn recruits Sir4, there is no clear evidence so far supporting a direct interaction between Abf1 and the SIR complex.

Interestingly, replication has been proposed to be required to establish silencing in *Saccharomyces cerevisiae* (Miller and Nasmyth 1984). However, this notion has been challenged by the observation that silencing can be established upon targeting of Sir1 to a non-replicative extrachromosomal cassette containing a crippled *HMR* locus (Kirchmaier and Rine 2001; Li et al. 2001). In this artificial context, silencing establishment retained cell cycle dependence, arguing that the passage of the replication fork is not required for establishment of yeast silent chromatin, although this does not exclude that it may contribute under some conditions. Interestingly, heterochromatin formation also shows cell cycle specificity in mammalian cells as microinjection of non-replicating plasmids in late S phase is transcriptionally repressed and associated with non-acetylated histones, whereas plasmid microinjected in early S phase is hyperacetylated and transcriptionally active (Zhang et al. 2002).

Although replication is not always necessary to establish silencing on non-chromosomal DNA, several observations indicate that fork pausing can contribute to heterochromatin establishment in budding yeast. First, it is noteworthy that natural sites of silencing (telomeres, *HM*, and ribosomal DNA (rDNA)) are all sites of transient replication fork pausing. Indeed, Rap1, ORC, Abf1, and Fob1 bind directly to the DNA and their tight interaction with their DNA-binding sequences acts as a replication roadblock (Brewer and Fangman 1988; Ivessa et al. 2003; Makovets et al. 2004). Interestingly, ORC binds more tightly at the *HMR-E* silencer element than at a very efficient replication origin found in euchromatin. Furthermore, this high-affinity binding site is required for ORC-dependent silencing at *HMR* (Palacios DeBeer et al. 2003). Second, we showed that natural or artificial pause sites can favor silencing establishment at ectopic loci (Dubarry et al. 2011). Importantly, this effect is increased in the absence of the DNA helicase Rrm3, which is known to facilitate replication at non-histone protein-DNA complexes. These data support a model where prolonged replication pausing increases the probability to form heterochromatin.

At endogenous loci, massive and stable SIR recruitment occurs only at sites harboring multiple DNA-protein complexes that have affinity for components of the SIR complex (i.e., Rap1 and Orc1). It is thus possible that replication fork pausing imposed by these complexes contributes to SIR recruitment at these sites, where they are then maintained by the affinities of Rap1 and Orc1 for Sir3, Sir4, and Sir1. Consistent with this hypothesis, SIR-dependent silencing of cryptic mating-type loci in *K. lactis* requires a different set of DNA-binding proteins for silencing establishment (Barsoum et al. 2010; Sjostrand et al. 2002). Further supporting this model is a recent report that a direct interaction between Sir3 and the ATPase subunit of the nucleosome remodeler Swi2 is key for SWI/SNF to promote resistance to replication stress in vivo and for the establishment of heterochromatin at telomeres (Manning and Peterson 2014).

In *Schizosaccharomyces pombe*, whose heterochromatin is more related to that of metazoans, mutations affecting the pool of dNTPS, thus slowing the replication fork and contributing to replication stress—favor the spreading of silent chromatin across the heterochromatin barriers of the silent mating-type locus mat2/3 (Singh and Klar 2008). Intriguingly, this extended heterochromatin depends on the binding of the transcription factor Atf1 that recruits histone deacetylases at this locus. Thus, the paradoxical role of transcription factors in heterochromatin formation in relation with replication stress appears as a recurrent theme.

In mouse and human genomes, the repetitive sequences associated with Suv39 H3K9me3-dependent heterochromatin contain transcription factor binding sites (Bulut-Karslioglu et al. 2012). Importantly, depletion of the two homeotic transcription factors Pax3 and Pax9 that bind mouse satellite repeats results in a derepression of these sequences and loss of heterochromatic marks. This led to a proposal that the reiterate arrangement of transcription factor binding sites in repetitive sequence is a general mechanism for heterochromatin formation (Bulut-Karslioglu et al. 2012). It will be interesting to test whether these factors represent obstacles for the replisome in this context.

What could be the function of heterochromatin formation at tight DNA-protein complexes sites? In *S. cerevisiae*, the histone deacetylase Sir2 represses PolII transcription and reduces recombination that would otherwise arise from the tight binding of Fob1 at rDNA repeats (Kobayashi 2011).

A similar function has been proposed in *S. pombe* for the CENP-B heterochromatin proteins that stabilize replication roadblocks imposed by the DNA-binding protein Sap1 at long terminal repeat retrotransposons (Zaratiegui et al. 2011b). Outside repetitive sequences, repressing transcription close to replication stress sites could contribute to genome integrity by preventing collision between the replication and transcription machineries.

### DNA secondary structures

DNA secondary structures such as hairpin or G-quadruplex (G4) pose specific threats to the progression of the replication machinery (Leon-Ortiz et al. 2014). G4 are four-strand DNA structures held together by guanine, which can be unwound by several conserved helicases in vitro (WRN, BLM, FANCJ, and PIF1). Telomere sequences are predicted to form G4 and were shown to pose a challenge for the replication machinery in the absence of the telomere binding factors Taz1 in fission yeast and telomeric repeat factor 1 (TRF1) in human cells (Miller et al. 2006; Sfeir et al. 2009). TRF1 possibly alleviates this replication problem by recruiting the BLM helicase (Sfeir et al. 2009). In human cancer cells, the G4 stabilizing molecule pyridostatin targets nuclear sites overlapping with hPif1 (Rodriguez et al. 2012). Furthermore, this drug promotes growth arrest via inducing replication- and transcription-dependent DNA damage at telomeric and non-telomeric sequences with a propensity to form G4, arguing that these structures do form in vivo.

Furthermore, G4 structures were shown to cause epigenetic instability in the absence of G4 processing enzymes in metazoan and yeast (Sarkies et al. 2012). In *S. cerevisiae*, inserting a G4 motif 40 kb away from telomere VL in Pif1-defective cells induces SIR-dependent epigenetic instability on reporter genes located on the telomere proximal flanking region (Paeschke et al. 2013). Along the same lines, DT40 chicken cells defective for FANCJ show increased chromatin compaction in active regions of the genome (Schwab et al. 2013). Furthermore, loss of gene expression and active chromatin marks occur in the vicinity of G4 structure in the absence of the translesion polymerase Rev1 (Schiavone et al. 2014).

Trinucleotide repeats are prone to genomic instability, and their expansion is a recurrent cause of several human diseases including Friedreich’s ataxia (GAA/TTC) and fragile X syndrome (CGG). In both cases, trinucleotide expansion in a non-coding region leads to the silencing of the surrounding locus. Studies in transgenic mice indicate that GAA-triplet expansions stimulate heterochromatinization of a neighboring gene (Saveliev et al. 2003), a process that can be reduced by inhibiting the Sir2 family of deacetylases (Chan et al. 2013). Mechanisms leading to heterochromatin formation at expanded trinucleotide repeats are not well understood. Given that trinucleotide repeats are prone to form hairpin secondary structures and interfere with replication, replication stress could trigger heteterochromatin formation at these sites (Mirkin 2007).

## Heterochromatin at replication/transcription collisions

Another source of replication stress arises from transcription—replication collisions (Azvolinsky et al. 2009). Such collisions seem to play a central role in the maintenance of pericentromeric heterochromatin in fission yeast. Genetic and biochemical evidence supports a model in which collisions between the replisome and the RNA polymerase II transcription complex generate stalled replication forks at these sites. It has been shown that, during replication, co-transcriptional RNA interference (RNAi) mechanisms release RNA polymerase II (Pol II) and avoid conflicts with the replication machinery (Li et al. 2011; Zaratiegui et al. 2011a). This is accompanied by the stable recruitment of the major silencing complex in fission yeast-cryptic loci regulator complex (CLRC) and the faithful propagation of heterochromatic states. The CLRC complex bridges these processes by interacting with subunits of the RNA-induced transcriptional silencing (RITS) complex as well as Cdc20 and Mms19, a subunit of the leading strand polymerase polε and a regulatory subunit of the Pol II transcription factor TFIIH, respectively (Bayne et al. 2010; Li et al. 2011; Motamedi et al. 2008; Svejstrup 2010). Zaratiegui and colleagues proposed that CLRC recruits the RNAi machinery at sites where the transcriptional and replication machineries clash. The RNAi machinery is able to remove Pol II and allow the resumption of DNA synthesis and further spreading of heterochromatin along the chromatin fiber (Kloc et al. 2008; Zaratiegui et al. 2011a). Whether this mechanism is conserved in other eukaryotes is not clear.

## Mechanisms linking replication stress with heterochromatin formation

If replication stress contributes to heterochromatin formation, one can wonder what prevents heterochromatin formation at coding euchromatic regions where replication stress is likely to occur. One possibility is that counteracting activities associated with transcription destabilize heterochromatin or prevent its formation. Interestingly, the William syndrome transcription factor (WSTF) was shown to interact with PCNA to target the chromatin remodeler Snf2h to replication forks, thus preventing heterochromatin formation at ectopic sites (Culver-Cochran and Chadwick 2013; Poot et al. 2004). Whether these sites correspond to sites of replication stress is not known.

Although the mechanism(s) linking replication stress with heterochromatin formation remains largely unknown, several conserved factors arise as good candidates to link these two processes. First, cohesin is a conserved and essential multiprotein complex that holds together newly replicated chromatids and plays a crucial role in the maintenance of genomic stability (Jeppsson et al. 2014). Cohesins were shown to accumulate at replication sites when DNA synthesis is impeded and are critical for the recovery of stalled forks in budding yeast (Tittel-Elmer et al. 2012) and possibly in human cells (Gatei et al. 2014). Importantly, both Sir2 in budding yeast and HP1/Swi6 in metazoan and fission yeast interact directly with cohesins (Nonaka et al. 2002; Wu et al. 2011), again supporting a model in which replication stress sites could stimulate the establishment of heterochromatin.

Replication stress, like DNA damage, triggers phosphorylation of serine 129 of histone H2A in yeast (γH2A) or the histone variant H2A.X in mammalian cells (Downs et al. 2000; Foster and Downs 2005; Rogakou et al. 1998). This modification aids chromatin remodeling and the recruitment of repair factors during DNA repair, but its role at replication-stalled sites is unclear. This modification is enriched at heterochromatic domains in both budding and fission yeast, and this depends, respectively, on the SIR complex and the histone methyltransferase Clr4 (Kim et al. 2007; Kitada et al. 2011; Rozenzhak et al. 2010; Szilard et al. 2010). Although enrichment of this mark could be a passive consequence of the low turnover of nucleosomes in heterochromatic regions, it has the potential to recruit specific factors.

In *S. pombe*, one of the factors that recognizes γH2A is the Brct containing domain protein Brc1 (Williams et al. 2010) that is recruited to pericentric heterochromatin during S phase. Interestingly, Brc1 mutants show defects in centromeric silencing and increased chromosome missegregation in the presence of a microtubule-destabilizing agent (Lee et al. 2013; Lee and Russell 2013). Genetic evidences suggest that Brc1 stabilizes the replisome in these regions, thus avoiding replication restart through the recombination machinery, which can lead to loss of genetic and epigenetic information. Again, although budding yeast heterochromatin has diverged from one of the *S. pombe*, the functional link between scaffolding repair proteins and heterochromatin appears to be conserved. Indeed, Brc1 resembles Rtt107/Esc4 in budding yeast, both structurally and functionally (Zappulla et al. 2006). Esc4 also interacts with γH2A and was recently shown to dampen checkpoint activation at replication-induced lesions (Ohouo et al. 2013). Intriguingly, Esc4 also interacts with the silencing factor Sir3 and mediates Sir3-dependent establishment of heterochromatin when targeted (Ohouo et al. 2010; Roberts et al. 2006; Rouse 2004; Zappulla et al. 2006). Whether this interaction plays a role in dampening checkpoint activation has not yet been tested. However, silent chromatin was shown to suppress the checkpoint response upon induction of massive replication fork blocks in the rDNA of *S. cerevisiae*, indicating that this mechanism could be conserved in budding yeast (Bentsen et al. 2013). Esc2 provides another potential molecular link between replication stress and silent chromatin. Like Esc4, Esc2 was first identified for its ability to establish silencing when targeted to a modified silencer, possibly through its ability to recruit Sir2 (Dhillon and Kamakaka 2000; Cuperus and Shore 2002). Esc2 is a protein conserved from yeast to human containing two SUMO-like domains (Yu et al. 2010) that play a role in the resolution of replication coupled recombination intermediates in conjunction with Smc5-Smc6 and Mms21 (Albuquerque et al. 2013; Choi et al. 2010; Mankouri et al. 2009; Mimura et al. 2010; Sollier et al. 2009).

More indirect mechanisms could also initiate heterochromatin formation at sites of replication stress. These include defects in recycling of parental histones and unscheduled histone incorporation that could lead to loss or gain of specific histone marks or histone modifications and remodeling associated with post-replicative repair events (Alabert and Groth 2012).

**Fig. 1:**
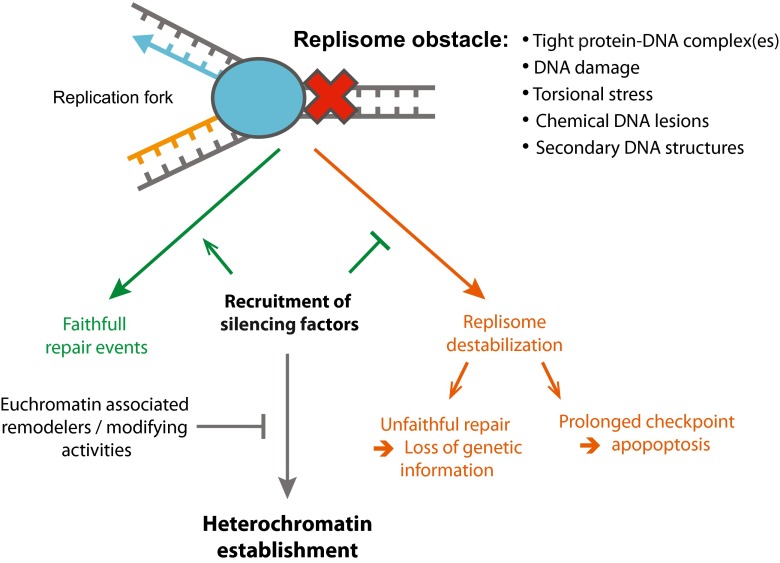
During S phase, the replisome encounters a number of impediments, which could interfere with its progression and have the potential to endanger the stability of the genome. When faced with such obstacles, in many cases, the cell triggers the S phase checkpoint and stabilizes the replication fork. In the event of replication fork breakdown or “collapse,” replication can restart by recruiting an alternative pathway requiring homology-directed repair (HDR), which could eventually result in loss of genetic and/or epigenetic information. If stalled forks fail to restart, persistent checkpoint activation can lead to apoptosis. As discussed in the text, silencing factors are also recruited to sites of replication stress. Possible roles of heterochromatin proteins at these loci could be in preserving the stability of the replisome or in modulating the cellular response to replication stress via largely unknown mechanism(s). Such events could also serve as an initial signal and potentially trigger the formation of heterochromatin

As mentioned above, some factors involved in DSB repair are also linked to gene silencing. It is tempting to speculate that DNA insults, irrespective of whether they occur during S phase or outside of it, could serve as an initial signal for the recruitment of silencing proteins with the capacity to induce the formation of heterochromatin structures. This local recruitment of silencing factors would likely be transient as shown for the recruitment of heterochromatin factors at euchromatic DNA damage sites (see above) and would thus not systematically lead to heterochromatin formation at most loci. However, one can speculate that the density of these events and/or the lack of antagonistic activities (i.e., histone remodeler of modifier) could lead to heterochromatin formation and maintenance at repetitive sequences. In these regions, heterochromatin formation would in turn avoid further stress by repressing transcription (avoiding replication/transcription collision), stabilizing the replication fork, and/or limiting checkpoint activation (Fig. 1).

## Concluding remarks

Many studies from different organisms have identified a crosstalk between replication stress and heterochromatin formation. It is striking that similar processes seem to be at play despite the un-conserved molecular nature of heterochromatin. This raises the question of the potential role(s) of this crosstalk. The following two recurrent themes emerge from the above-discussed studies (Fig. 1): heterochromatin dampening the checkpoint activation at replication stress sites and heterochromatin stabilizing the replisome by avoiding collision between the replication and transcription machineries or by avoiding unscheduled recombination events. On the other hand, the numerous links between replication stress and heterochromatin strongly indicate that replication stress could be an evolutionary conserved auxiliary mechanism for the establishment of heterochromatin and silencing domains. Further studies are needed to unravel the candidates and cascade of events that govern this complex crosstalk.
